# Can a person-centred-care intervention improve health-related quality of life in patients with head and neck cancer? A randomized, controlled study

**DOI:** 10.1186/s12912-017-0206-6

**Published:** 2017-02-21

**Authors:** Elisabeth Hansson, Eric Carlström, Lars-Eric Olsson, Jan Nyman, Ingalill Koinberg

**Affiliations:** 1 The Sahlgrenska Academy—Institute of Health and Care Sciences, University of Gothenburg, Gothenburg, Sweden; 2 Centre for Person-Centred Care (GPCC), Gothenburg University, Gothenburg, Sweden; 3 Department of Oncology, Sahlgrenska University Hospital, Gothenburg, Sweden; 4 University College of South East Norway, Notodden, Norway

## Abstract

**Background:**

The incidence of head and neck cancer is increasing slightly. Head and neck cancer but also it’s necessary and often successful treatment may affect general domains of health-related quality of life and provoke a variety of adverse symptoms and side effects, both during and after treatment. The objective of this study was to compare a person-centred care intervention in terms of health-related quality of life, disease-specific symptoms or problems, with traditional care as a control group for patients with head and neck cancer.

**Methods:**

In this randomized controlled trial, person–centred-care intervention and traditional care (control) groups comprised 54 and 42 patients, respectively. Outcome measures used were: the EORTC QLQ-C30 and the EORTC QLQ-C35. Both groups answered the questionnaires at baseline and after 4, 10, 18 and 52 weeks from start of treatment. The questionnaires’ scores were compared between groups by using independent samples test and non-parametric test for continuous variables. For categorical data, Fisher’s exact test was used. Longitudinal data were analysed using generalized linear models for normally distributed repeated measures data.

**Results:**

At baseline, the intervention and control groups were comparable in terms of medical and sociodemographic variables, clinical characteristics, health-related quality of life and disease-specific symptoms or problems. At all the follow-up points, even during the worst period for the patients, the person-centred-care group consistently reported better scores than the control group. The differences were numerically but not always statistically significant. When testing longitudinal data, statistically significant results were found for head and neck cancer-specific problems, swallowing (*p* = 0.014), social eating (*p* = 0.048) and feeling ill (*p* = 0.021).

**Conclusions:**

The results from this study suggest that adopting the person-centred-care concept practiced here could be a way to improve function and wellbeing in patients with head and neck cancer.

**Trial registration:**

The study was retrospectively registered in 2016-12-05 in Clinical Trials gov. “Can a Person-centred-care Intervention Improve Health-related Quality of Life in Patients With Head and Neck Cancer” registration number: NCT02982746.

## Background

Annually, approximately 1400 new cases of head and neck cancer (HNC) are diagnosed in Sweden. The incidence of HNC is increasing slightly, especially for oropharyngeal carcinoma due to human papilloma virus (HPV). Long-term survival varies with the sub site, but the disease-specific survival rate in general is approximately 60%. The tumours often affect vital functions causing eating, breathing and communication symptoms. They also influence speech, sight and hearing abilities, as well as body image and appearance [1]. HNC but also it’s necessary and often successful treatment may affect general domains of health-related quality of life (HRQoL) and provoke a variety of adverse symptoms and side effects, both during and after treatment. Symptoms such as severe mucosal reactions, nausea, dysphagia xerostomia and taste changes cause nutritional problems and persist usually throughout the treatment regime and then gradually improve during the first year after treatment [2–4]. Other frequently experienced symptoms and side effects such as pain, vomiting and fatigue during extended periods are all problems that are lowering quality of life contributing to social negative effects like prolonged work ability and in the long run also the survival [5–7]. Due to the tumour’s localization, the symptoms usually lead to eating difficulties and the patients usually need enteral feeding via a PEG (Percutaneous Endoscopic Gastrostomy) or NG (Nasogastric tube).

Patients affected by HNC have traditionally been described as a vulnerable group with a poor prognosis, and the diagnosis is often associated with tobacco and alcohol abuse. The disease usually affects older men (median age 66 years) and, to some extent, individuals from a lower socio-economic background. The increase in the incidence of oropharyngeal cancer has been linked to HPV infection [8]. Notably, patients with HPV-positive oropharyngeal cancer are younger than the average age of people affected by HNC (median age 62 years) and are most often non-smokers [9, 10]. Earlier studies have shown that patients with HNC experience deterioration in HRQoL during and up to a year after treatment [2, 11]. The rates of depression related to the physical symptoms have also been found to increase among patients suffering from HNC [12–14]. The suicide rates for people affected by HNS are substantially higher than in both the general population and the overall population of patients with cancer [15].

The national health-care system serving patients suffering from such complex and multifaceted diseases as HNC has been characterized by an insufficient co-ordination and continuity of care and treatment [16]. In the case of patients with HNC in Sweden, there are too few outpatient clinics and the primary-care centres are not able to treat these patients sufficiently well. This is a well-known scenario for certain types of diagnoses resulting in a lack of continuity in the transition of patients between hospital and primary care [16–19]. Today there is nobody among the professionals who coordinates the care. In teams focusing on person-centred care (PCC), the patients are seen as partners and therefore as a member of the team [20]. The rationale for this is that knowledge of the patient’s everyday life, abilities and perceptions is regarded as the key to planning care in partnership with the patient [21]. Patients are expected to be actively involved in both the planning and execution of the care and rehabilitation process. A PCC philosophy defined as Gothenburg person-centred care (gPCC) emphasizes that the patient’s own resources can make the difference between a successful and a less successful rehabilitation process [21]. gPCC teams have been shown to be capable of successfully combining high-quality health care and focusing on the patient’s own goals [21–23].

Consequently, there is a need to evaluate whether a person-centred team intervention can improve symptom control with preserved function and quality of life.

The aim of this study was to compare a person-centred care intervention in terms of HRQoL related to disease-specific symptoms or problems, with traditional care as a control group for patients with HNC.

## Methods

### Study design

We conducted a single-centre, randomized, longitudinal controlled study with an intervention group (gPPC-G) and a control group (CG). The study was performed between the spring of 2012 and the spring of 2014 at Sahlgrenska University Hospital in Sweden.

### Participants

The incidence of HNC in the hospital’s catchment area is around 260 patients per year, approximately 20% of the incidence in the entire country. Eligible for this study were patients diagnosed with HNC, older than 18 years and able to read and write Swedish. The patients should be suitable for outpatient treatment with chemo- and/or radiotherapy, either as primary treatment or in a postoperative setting, decided of an oncologist. Patients were excluded if they had a previous or concomitant malignancy or were diagnosed and treated for depression as stated in their medical record. To avoid influences from other studies, none of the patients were to be included if they were taking part in other research studies.

### Power analysis and randomisation

Power calculation was performed based on the EORTC QLQ-30 instrument and it showed that around 100 patients were needed to achieve 80% power to detect a 20% difference at the 5% significance level (mean value of 63 and SD 23,9 for General Quality Of life, EORTC QLQ30 instrument) [24].

**Fig. 1:**
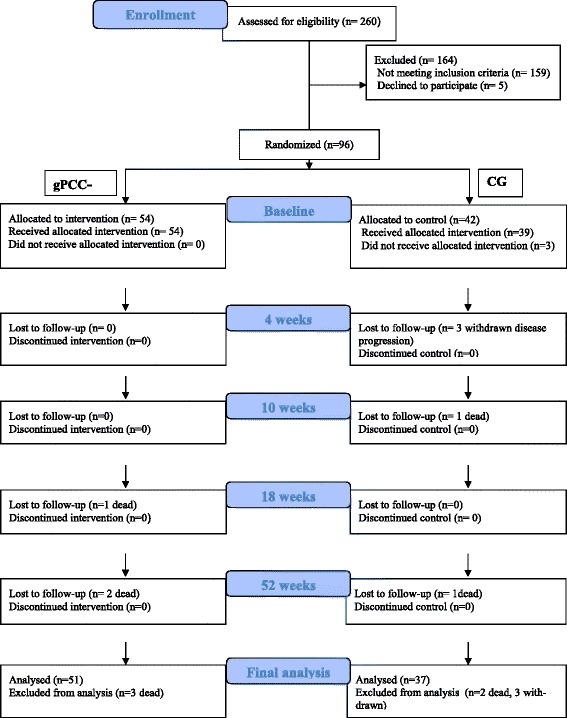
CONSORT DIAGRAM, flow chart of the participants in the gPCC—G and the CG

A total of 101 patients were identified as eligible for inclusion (Fig. 1). The study was explained and patients were asked by the oncologist to participate. Five patients declined to participate. Both written and verbal informed consent was thus obtained from 96 consecutively selected patient diagnosed with HNC classified in stages I–IV, they were randomized to either the gPCC-G or the CG (Fig. 1). The randomization of the patients took place at the outpatient clinic in connection with the first meeting with the oncologist. Because this particular type of intervention had never been attempted with this patient group, and since we did not expect any negative effects of the intervention, we assumed that the intervention group needed to be larger than the control group. Randomization was conducted and handled by the study nurse, as a computerized randomization process and using sealed opaque envelopes, assigning 60% of the patients to the intervention group (*n* = 54) and 40% to the control group (*n* = 42) (Fig. 1). Due to practical reasons no one was blinded after randomisation.

### Treatment procedure

With a few exceptions, all of the patients being treated at the hospital are routinely discussed and assessed at a weekly multidisciplinary tumour board. Thereafter, the physician discusses the treatment options with the patients and their relatives and, for the few patients not participating in the multidisciplinary treatment conference, this discussion is held at a scheduled meeting with the oncologist. The treatment, which includes chemotherapy and radiotherapy according to the chosen treatment strategy, usually starts within 2 weeks. During the treatment period, which often lasts up to 6 weeks, the patients are seen at weekly consultations with a physician at the radiotherapy department. After the treatment, normally after a period of 6 to 8 weeks, the follow-up routines include an initial return visit, followed by a visit every 3 months for 2 years, preceded by a radiological evaluation. After discharge from the clinic, the patients are referred back to their local hospitals or their primary-care centres for support in the event of any complications that might arise. No follow-up appointments with nurses are usually made, but the patients are given a phone number they can call if needed.

### Control group (CG)

Patients randomized to the control group received usual care and return visits were scheduled according to the treatment procedure described under the previous heading and based on the Regional care program for patients with HNC [25]. CG patients were recruited at the same time and in the same way as those in the intervention group.

### The gPCC intervention group (gPCC-G)

Patients randomized to the intervention group were contacted and scheduled to attend a meeting at the oncology clinic with the nurse specialist in oncology, accompanied by a close relative, if possible, within 7 days after their first visit to the oncologist. This first meeting included a description of the study as well as information needed about the health-care plan. The plan was designed and developed according to a basic model from gPCC and further adapted to suit patients with HNC and scheduled by the nurse and patient together. Furthermore, the nurse informed the patients and relatives, orally and in writing, about symptoms and side-effects known to have an impact on quality of life, such fatigue, nutrition problems, weight loss, pain and nausea. The patients were asked to describe their life situation (work, leisure time, stress, family and social life, social support and potential problems), experiences regarding diagnosis and treatment, and prior experience relating to nausea and pain. As needed, other factors associated with lifestyle (diet, smoking, alcohol consumption, mental health etc.) were discussed. The patient and the nurse shared information by reviewing the medical record relating to previous blood tests, X-ray examinations, chemotherapy, radiotherapy, use of medications and the validation of symptoms such as pain and fatigue. Each patient also described her/his eating problems and treatment strategies were advised and discussed. In this partnership, factors in the patient’s narrative which were considered crucial for addressing eating problems, such as weight loss, nausea and pain, were identified, clarified and concretized. Patient involvement in planning was emphasized. The treatment process was discussed to suit each person’s circumstances as a part of the health-care plan. The patient’s preferences, goals and indications for and barriers to the impending treatment, including personal, environmental and social factors, were discussed. An important factor taken into account was to ensure that symptoms and side effects of treatment would not compromise the perceived balance between life activities. The health-care plan comprised self-management goals that were formed in partnership between the patient and the nurse. Each patient was encouraged to reflect on their self-management goals, how to reach them, and to anticipate barriers; and to refine the plan. The health plan includes both short- and long-term goals for the patient along with the actions needed to reach each goal. The plan is a “living” document specific to each patient, in which the goals and actions are tracked and revised over time. Because the treatment greatly affects the patient’s quality of life and ability to eat, the plan encourages constant discussion about whether the patient is managing these symptoms and side effects, or whether an NG or PEG is needed or whether hospitalization is necessary. The patient was also given a direct telephone number to reach the nurse specialist if they had any questions about anything relating to their treatment and wellbeing. The nurse documented the health-care plan in the medical record.

### Data collection and measurement

The patients answered the first set of questionnaires either in the hospital after the multidisciplinary treatment conference or at home. The remaining sets of questionnaires were answered at 4, 10, 18 and 52 weeks after the start of treatment. The oncologists’ clinical knowledge was that the time points chosen typically represented significant but different clinical symptom stages during of the treatment. The questionnaires were returned by the patients by post, in a prepaid envelope. The survey consisted of two outcome measurements: the European Organization for Research and Treatment of Cancer (EORTC) QLQ-C30 and the EORTC QLQ-35 version 3.0.

The EORTC QLQ-C30 is a cancer-specific instrument that has been developed for measuring HRQoL in clinical trials and is widely used. It has a good psychometric property for use with patients affected by HNC. The questionnaire consists of five sub-scales; physical function, role function, emotional function, cognitive function and social function, three symptom scales (nausea, pain and fatigue), global health status (GHS), and six single-symptom items, making a total of 30 questions. All scores result in a value between 0 and 100; high scores on functional and global quality of life imply a high level of function, while a higher score on the symptom scales indicate greater problems [3, 24, 26–28]. In the present study, we have focused on the five subscales, one symptom scale (pain) and the GHS.

The EORTC QLQ-35 is a cancer-specific instrument developed to measure common symptoms related to patients with HNC. The instrument comprises 35 questions, seven multiple-item scales that assess the symptoms of pain, swallowing ability, senses (taste/smell), speech, social eating, social contact, sexuality and six single-item scales, which survey the presence of symptomatic problems associated with teeth, mouth-opening, dry mouth (xerostomia), sticky saliva, coughing, and feeling ill. All scales and single items are scored from 0 to 100; a high score corresponds with a high level of symptoms or problems. A Swedish version of the instrument appears to be reliable, valid and applicable to broad multicultural samples of patients with HNC [29]*.*

In the current study we have replaced the questions about feeding tube, nutritional support and weight loss with those related to similar issues but framed within the study context: “Weight loss changes in percent from baseline to study completion”, “feeding tube or PEG during the treatment period” and “used nutritional support during the treatment period”.

The EORTC QLQ-35, in conjunction with the EORTC QLQ-30, can be regarded as a standard instrument to measure HRQoL and diagnose specific symptoms in patients with HNC [24, 30].

For all patients, their sociodemographic and medical data were gathered and documented based on those recorded in their medical record (Table 1).

**Table 1:** Baseline characteristics for the intervention group (gPCC-G) and the control group (CG)

Variable	gPCC-G *n* = 54	CG *n* = 42
Age Mean (at baseline) (SD)	61 (7.8)	62 (10.5)
Male (%)	70	69
Employed (yes) (%)	67	41
Living with someone (%)	80	88
Tumor site: (%)		
Oropharynx	65	76
Larynx	11	14
Neck node with unknown primary	6	5
Hypopharynx	6	0
Oral cavity	4	5
Parotid gland	4	0
Paranasal sinus	2	0
Nasopharynx	2	0
Clinical stage: (%)		
I	5	12
II	13	7
III	19	19
IV	63	62
Treatment: (%)		
Chemo radiotherapy	72	76
Primary radiation	28	24
HPV positive (%)	68	62
HPV negative (%)	13	26
HPV not known (%)	19	12

Values are mean, standard deviation (SD) and %

*HPV* Human papillomavirus

### Statistical Analysis

Analyses for this trial were completed on an intention-to-treat basis and data from all the individuals who entered the trial and answered the questionnaires were analysed by initial group assignment. Demographic data were expressed as number (*n*), percent (%), mean and standard deviation (SD) and 95% confidence interval (CI). The EORTC QLQ 30 and EORTC QLQ 35 scores were compared between groups by using independent samples test (Student’s *t*-test) and non-parametric test (Mann-Whitney *U* Test) for continuous variables. For categorical data, Fisher’s exact test was used.

The longitudinal data were analysed by using generalized linear models for normally distributed repeated measures data with treatment group, time and interaction between treatment group, and time as fixed effects and baseline value as covariate. The generalized linear model used in all models was compound symmetry.

Least Square Means with associated 95% CI are presented for both groups and for the difference between the two groups and the corresponding *p*-value for the difference.

All tests were two-tailed and conducted at 0.05 significance level.

SPSS version 21 and SAS Software (version 9.4) were used for statistical calculations.

## Results

Data were obtained from 88 of the 96 persons treated for HNC. Of the eight patients who did not complete all the questionnaires, three were from the CG (withdrawn after consent and disease progression < 4 weeks after the start of the treatment). Five patients died during the study; three from the gPCC-G (one patient between second and third follow up and two patients between third and fourth follow up) and two from the CG (one patients between baseline and the first follow up and one patient between third and fourth follow up) (Fig. 1). The internal response rate varied somewhat between the measurement points, from 93% (gPCC-G) and 86% (CG) at baseline, to 78% (gPCC-G) and 69% (CG) at 4 weeks, 82% (gPCC-G) and 71% (CG) at 10 weeks, 87% (gPCC-G) and 67% (CG) at 18 weeks, and 85% (gPCC-G) and 69% (CG) at 52 weeks. No statistically significant differences between the groups were found in this respect—except at the 18-week measurement point, when the response rate in the intervention group was higher (*p* = 0.024).

All patients in the intervention group received person-centred care; no one refused such treatment during the study period.

**Fig. 2:**
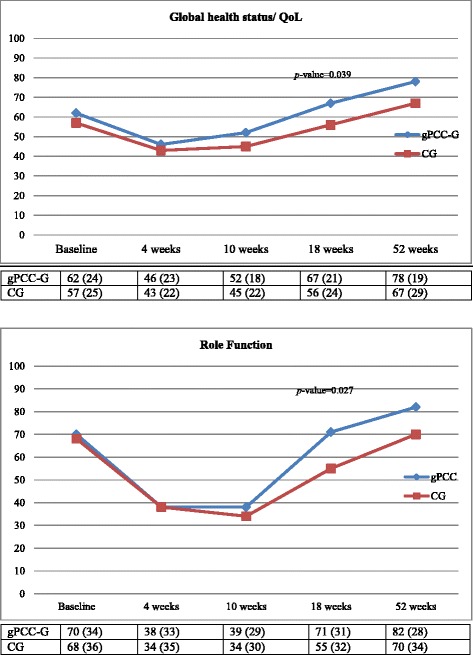
Mean values and standard deviation (SD) of the Global Health status and Role Function (QLQ-30) sub-scores at the five the measurement points, for both groups

**Fig. 3:**
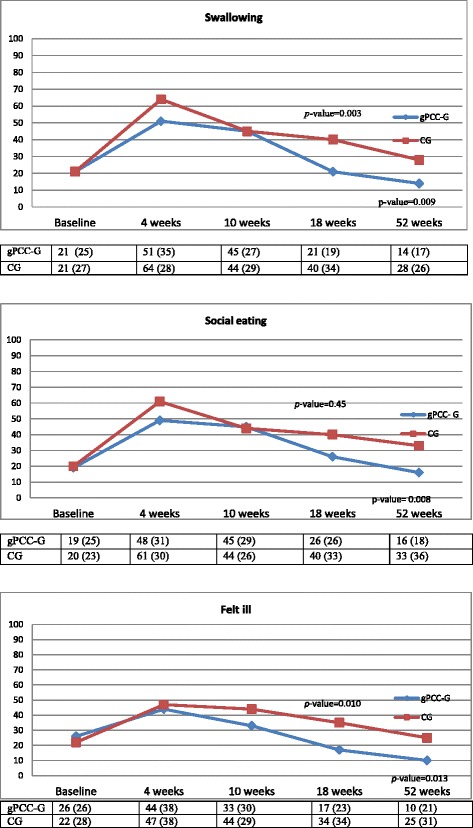
Mean values and standard deviation (SD) of the Swallowing, Social eating and Felt ill (QLQ-35) sub-scores at the five measurement points, for both groups

At baseline, the gPCC-G and the CG were comparable in terms of sociodemographic variables, clinical characteristics, HRQoL and disease-specific symptoms (Table 1 and Figs. 2 and 3).

During the follow-up year the internal response rates and pattern of change in the scores over time were strikingly similar in both the groups (Figs. 2 and 3). Between the 4th to 10th week measuring points, all the scales were at their lowest or highest, indicating that this was the time period during which the patients experienced the worst HRQoL. After this deterioration, both groups gradually improved to levels that, at least for the gPCC-G, were better than at baseline (Figs. 2 and 3). From 4 weeks on, the gPCC-G reported numerically higher scores. When the sub-scales were tested with a cross-sectional analysis (at each measurement point), statistically significant differences between the two groups were found for most of the sub-scales at the 18th and/or 52nd week’s measurement points (Figs. 2 and 3), both for HRQoL and HNC-specific problems.

When the analysis was advanced by using generalized linear models for normally distributed repeated measures data a similar pattern as that found in the cross-sectional analysis was revealed, that is to say, the results in the gPCC-G tended, from the 10th week, to be better than those in the CG and were, from the 18th week, statistically significantly better in the gPCC-G in terms of HNC-specific problems (QLQ-35), swallowing (*p* = 0.014), social eating (*p* = 0.048) and feeling ill (*p* = 0.021) (Table 3). The gPCC-G reported numerically higher scores but these were not significantly better than those reported by the CG in terms of HRQoL (QLQ-30) (Table 2).

**Table 2:** Results from generalized linear models for normally distributed repeated measures data for QoL- 30 variables including corresponding baseline value as covariate

	Week 4			Week 10			Week 18			Week 52		
Variable	Intervention	Control	Difference Intervention- Control	Intervention	Control	Difference Intervention- Control	Intervention	Control	Difference Intervention- Control	Intervention	Control	Difference Intervention- Control
Quality of Life	45.0 (38.5–51.4)	46.7 (38.9–54.6)	−1.7 (−11.9–8.4) *p* = 0.74	49.5 (43.2–55.9)	47.8 (40.2–55.5)	1.7 (−8.3–11.7) *p* = 0.74	66.2 (59.9–72.4)	58.6 (50.8–66.3)	7.6 (−2.4–17.6) *p* = 0.13	76.1 (69.9–82.4)	67.9 (60.2–75.7)	8.2 (−1.8–18.2) *p* = 0.11
Physical Function	69.2 (63.6–74.7)	68.6 (61.8–75.3)	0.6 (−8.2–9.4) *p* = 0.89	70.9 (65.4–76.4)	70.9 (64.3–77.4)	0.0 (−8.6–8.6) *p* = 1.00	82.7 (77.4–88.0)	78.9 (72.2–85.5)	3.8 (−4.7–12.3) *p* = 0.38	88.0 (82.6–93.4)	84.8 (78.2–91.5)	3.2 (−5.4–11.7) *p* = 0.47
Role Function	36.2 (26.6–45.8)	37.0 (25.4–48.7)	−0.9 (−16.0–14.3) *p* = 0.91	35.2 (25.7–44.8)	38.0 (26.6–49.4)	−2.7 (−17.6–12.2) *p* = 0.72	70.4 (61.1–79.6)	56.7 (45.1–68.2)	13.7 (−1.1–28.6) *p* = 0.070	81.2 (71.9–90.5)	75.3 (63.7–86.8)	5.9 (−8.9–20.8) *p* = 0.43
Emotional Function	72.5 (66.2–78.8)	64.0 (56.4–71.7)	8.4 (−1.5–18.4) *p* = 0.096	71.7 (65.4–77.9)	67.2 (59.7–74.7)	4.5 (−5.3–14.3) *p* = 0.37	76.3 (70.3–82.4)	68.1 (60.5–75.7)	8.2 (−1.5–17.9) *p* = 0.097	81.6 (75.5–87.7)	77.7 (70.1–85.3)	3.8 (−5.9–13.6) *p* = 0.44
Cognitive Function	72.5 (66.0–78.9)	65.7 (57.8–73.6)	6.7 (−3.6–17.0) *p* = 0.20	75.4 (69.0–81.7)	70.2 (62.5–77.9)	5.1 (−4.9–15.2) *p* = 0.31	83.7 (77.5–89.9)	79.0 (71.2–86.7)	4.7 (−5.3–14.7) *p* = 0.35	86.1 (79.9–92.3)	83.7 (76.0–91.5)	2.4 (−7.6–12.4) *p* = 0.64
Social Function	47.4 (39.1–55.8)	50.7 (40.6–60.9)	−3.3 (−16.4–9.9) *p* = 0.62	56.7 (48.4–64.9)	54.4 (44.5–64.2)	2.3 (−10.6–15.2) *p* = 0.73	77.1 (69.1–85.0)	67.9 (57.9–77.9)	9.2 (−3.6–22.0) *p* = 0.16	86.0 (78.0–94.1)	75.6 (65.6–85.7)	10.4 (−2.4–23.2) *p* = 0.11
Pain	49.0 (40.8–57.2)	58.0 (48.0–68.0)	−8.9 (−21.9–4.0) *p* = 0.17	39.5 (31.4–47.7)	41.4 (31.7–51.1)	−1.9 (−14.6–10.8) *p* = 0.77	21.3 (13.4–29.1)	30.1 (20.3–40.0)	−8.9 (−21.5–3.7) *p* = 0.17	17.6 (9.6–25.5)	25.2 (15.3–35.0)	−7.6 (−20.3–5.0) *p* = 0.24

All results are obtained by using generalized linear models for normally distributed repeated measures data with treatment group, time number and interaction between treatment group and time number as fixed effects and baseline value as covariate. The generalized linear model used in all models is compound symmetry

Least Square Means with associated 95% CI are presented for both groups and difference between the two and *p*-value for the difference

There were no differences found between the groups at any of the follow-ups (cross-sectional analysis) for specific problems such as senses of smell and taste, teeth, opening mouth, dry mouth, sticky salvia and the use of pain-killers (QLQ 35).

All patients in both the groups lost weight during the follow-up year. The average loss was 9.8% for the gPCC-G and 8.1% for the CG.

Almost 80% in both groups were supplied with a feeding tube or a PEG at least once during the follow-up year.

## Discussion

To survive their cancer, patients affected by HNC must undergo a very challenging treatment regime. The associated side effects of this treatment, such as pain, difficulties with food intake, loss of taste, dry mouth and risk of weight loss, can lead to the patient’s whole existence becoming seriously and adversely affected. To support and assist the patients during this treatment period, gPCC has been tested and compared with traditional care.

The main result in this study is that the response pattern and the progress during the follow-up year were quite similar in both groups. However, at all of the follow-up points the gPCC-G consistently reported better scores than the CG. The differences were numerically but not always statistically significant (Figs. 2 and 3 and Tables 2 and 3). All of the scales, both from the QLQ-30 and the QLQ-35 noted the lowest or highest (worst) value at the 4-week measurement point, indicating that this was the time period during which the patients experienced as the worst HRQoL fairly consistently. Murphy [12] reported a similar pattern, with a decline in HRQoL immediately after the start of therapy and a return toward baseline values after 1 year in patients with HNC. After the consistent decline noted in both the groups in the current study, the subsequent gradual improvement, at least for the gPCC group, was higher than for the baseline values (Figs. 2 and 3). A Cochrane review by Semple [31] concluded that there was no evidence to suggest that psychosocial intervention promotes global quality of life for patients with HNC at the end of the intervention. The only study we found with a comparable intervention was conducted by van der Meulen [32], who had a reactive approach in their intervention, with the emphasis on discussing problems and giving information and advice. In our study, we have a proactive approach with a person-centred focus. The proactive approach was based on the initial patient history and partnership, which resulted in a health plan where the patient’s individual needs were documented. Together with continuity and accessibility, these were the key components of the gPCC intervention and this created safe, supportive care.

**Table 3:** Results from generalized linear models for normally distributed repeated measures data for QLQ-35 variables including corresponding baseline value as covariate

	Week 4			Week 10			Week 18			Week 52		
Variable	Intervention	Control	Difference Intervention- Control	Intervention	Control	Difference Intervention- Control	Intervention	Control	Difference Intervention- Control	Intervention	Control	Difference Intervention- Control
HN Pain	48.4 (41.0–55.8)	50.8 (41.9–59.7)	−2.4 (−14.0–9.1) *p* = 0.68	38.8 (31.4–46.1)	38.4 (29.6–47.2)	0.3 (−11.1–11.8) *p* = 0.95	22.6 (15.4–29.8)	30.4 (21.4–39.3)	−7.8 (−19.3–3.7) *p* = 0.18	15.4 (8.1–22.6)	17.1 (8.2–26.0)	−1.8 (−13.3–9.8) *p* = 0.76
HN Swallowing	54.0 (45.9–62.1)	63.5 (53.9–73.2)	−9.5 (−22.1–3.1) *p* = 0.14	46.2 (38.1–54.2)	40.3 (30.7–50.0)	5.8 (−6.8–18.4) *p* = 0.36	21.2 (13.4–29.0)	36.8 (27.1–46.4)	−15.6 (−28.0–−3.2) *p* = 0.014	12.8 (4.9–20.6)	19.9 (10.3–29.6)	−7.2 (−19.6–5.3) *p* = 0.26
HN Speech	41.5 (33.3–49.6)	45.0 (35.4–54.5)	−3.5 (−16.1–9.1) *p* = 0.58	29.4 (21.2–37.5)	33.3 (23.7–42.9)	−3.9 (−16.6–8.7) *p* = 0.54	19.6 (11.7–27.5)	25.9 (16.2–35.6)	−6.3 (−18.9–6.2) *p* = 0.32	12.3 (4.2–20.3)	18.9 (9.2–28.6)	−6.6 (−19.2–6.0) *p* = 0.30
HN Social eating	51.5 (43.0–60.0)	60.4 (50.0–70.8)	−8.9 (−22.3–4.6) *p* = 0.19	46.3 (37.8–54.8)	40.1 (30.2–50.1)	6.2 (−6.9–19.3) *p* = 0.35	25.4 (17.2–33.5)	38.5 (28.4–48.6)	−13.1 (−26.1–−0.1) *p* = 0.048	16.1 (7.9–24.3)	26.3 (16.2–36.3)	−10.1 (−23.2–2.9) *p* = 0.13
HN Social contact	25.3 (18.4–32.3)	30.7 (22.5–39.0)	−5.4 (−16.2–5.4) *p* = 0.32	20.9 (14.0–27.8)	21.6 (13.4–29.7)	−0.6 (−11.4–10.1) *p* = 0.91	10.0 (3.3–16.7)	11.8 (3.6–20.1)	−1.9 (−12.6–8.8) *p* = 0.73	6.9 (0.2–13.7)	14.0 (5.7–22.3)	−7.1 (−17.8–3.6) *p* = 0.19
HN Coughed	43.3 (34.1–52.4)	40.2 (29.2–51.2)	3.1 (−11.4–17.6) *p* = 0.68	43.3 (34.3–52.3)	37.1 (26.3–48.0)	6.1 (−8.2–20.4) *p* = 0.40	25.7 (16.9–34.5)	35.6 (24.6–46.6)	−9.9 (−24.1–4.4) *p* = 0.17	15.0 (6.2–23.8)	21.8 (10.8–32.8)	−6.8 (−21.1–7.5) *p* = 0.35
HN Felt ill	46.2 (37.3–55.2)	46.2 (35.4–56.9)	0.1 (−13.9–14.1) *p* = 0.99	32.4 (23.5–41.2)	43.0 (32.4–53.6)	−10.6 (−24.4–3.2) *p* = 0.13	17.5 (8.9–26.1)	33.8 (23.0–44.6)	−16.3 (−30.1–−2.4) *p* = 0.021	8.9 (0.2–17.7)	21.3 (10.5–32.1)	−12.4 (−26.2–1.5) *p* = 0.080

All results are obtained by using generalized linear models for normally distributed repeated measures data with treatment group, time number and interaction between treatment group and visit number as fixed effects and baseline value as covariate. The generalized linear model used in all models is compound symmetry

Least Square Means with associated 95% CI are presented for both groups and difference between the two and *p*-value for the difference

Our results are in agreement with the findings in another study by van Der Meulen et al [33]. In their study, a nurse-led intervention had beneficial effects on some symptoms, for example pain and swallowing. In the present study, symptoms such as swallowing, social eating and feeling ill were significantly better in the gPCC-G, even when using repeated measures in covariance pattern models. However, in the current study, we could not see any statistical difference in terms of weight loss, as all patients in both the gPCC- G and the CG lost weight during the follow-up year. The average loss was 9.8% and 8.1%, respectively. One explanation could be that tumor location, stage and performance status and presence of dysphagia influenced the individual symptoms that predict impaired food intake. Almost 80% in both the groups were supplied with a feeding tube at least once during the follow-up year, and this did not affect the participants’ perceptions of their general or disease-related quality of life.

Van der Muelen [32] showed that a nurse-led psychosocial intervention had a long-term effect on HRQoL, particularly in terms of emotional and physical functioning, pain and depressive symptoms. The development of all outcome measurements in the current study generally corresponded with what has been noted in earlier longitudinal observational studies [24, 32, 34]. Although not always statistically significantly different, the results for the gPCC-G consistently tended to be better than those in the CG. One plausible explanation for the positive effect of the gPCC intervention on HRQoL, noted in earlier studies, could be the person-centred assessment and the health plan developed by the nurses and the patients together [23, 35]. The reason why the health plan does have such an effect is probably because it is developed based on the individual patient’s self and that the health plan is subjected to continuous revision if needed. The continuity with the specialised oncology nurses which was an important part of the intervention and the health plan probably also contributed. In a previous paper, we described how patients in both groups showed a high degree of trust in the cancer clinic [16]. According to usual practice, if problems developed, the patients in the control group were asked primarily to contact the health-care centre. This frequently resulted in a feeling of being abandoned after discharge, which, to a great extent, explained the increasing distrust shown by the control group, while the opposite was noted in the intervention group [16].

The limitations of our study include the fact that a number of patients did not respond to all of the questionnaires. The internal response rate was lowest at 4 and 10 weeks. It is possible that having a greater number may have revealed differences not distinctly verified here. However, when using the longitudinal data analysis, the missing data are handled. The study was completed on an intention-to-treat basis. A per-protocol-analysis would have needed a detailed information on how well each patient had completed the care plan, information not included in this study. All patients in the intervention group received person-centred care; no one refused such treatment during the study period, but as could be expected in their own personal way.

The study took place at just one centre, but included a large catchment area, which might restrict the generalizability of the findings. Contamination or dissemination of the intervention to the control group which could be a risk for a one centre study was unlikely since the patients were outpatients with small chances to meet.

With a well-functioning outpatient service for this special group of patients it cannot be ruled out that the current intervention may have achieved other results.

## Conclusion

The data indicate that PCC is a promising way to care for patients with HNC in order to improve their function and well-being. The treatment period from between the 4th and 10th week was the time period during which the patients experienced the worst HRQoL fairly consistently. After this period the patients gradually improved. At all the follow-up points, the gPCC-G tended to feel better than the CG and reported better QoL from the 18th week with almost no exception numerically, but not always statistically significantly better. This result implies that the gPCC concept was a health status-improving substitute that at least partly compensated for the negative effects of missing co-ordination and continuity of care.

### Relevance to clinical practice

The findings in this study suggested that the PCC concept should be included not only in clinical practice but also in nursing education.

## Abbreviations

EORTC QLQ-30: EORTC QLQ-34, European Organization for Research and Treatment of Cancer
gPCC: Gothenburg person-centred care
HNC: Head and neck cancer
HPV: Human papilloma virus
HRQoL: Health-related quality of life
NG: Nasogastric tube
PCC: Person-centred care
PEG: Percutaneous endoscopic gastrostomy
